# Trypanocidal Activity of Long Chain Diamines and Aminoalcohols

**DOI:** 10.3390/molecules200611554

**Published:** 2015-06-23

**Authors:** Ana L. Legarda-Ceballos, Esther del Olmo, Julio López-Abán, Ricardo Escarcena, Luis A. Bustos, Cristina Fonseca-Berzal, Alicia Gómez-Barrio, Juan C. Dib, Arturo San Feliciano, Antonio Muro

**Affiliations:** 1 Laboratory of Molecular Immunology and Parasitology, Faculty of Pharmacy, CIETUS, IBSAL, Universidad de Salamanca, 37007-Salamanca, Spain; E-Mails: analegarda@usal.es (A.L.L.-C.); jlaban@usal.es (J.L.-A.); ama@usal.es (A.M.); 2 Department of Pharmaceutical Chemistry, Faculty of Pharmacy, CIETUS, IBSAL, Universidad de Salamanca, 37007-Salamanca, Spain; E-Mails: ricar@usal.es (R.E.); asf@usal.es (A.S.F.); 3 Department of Pharmaceutical Sciences, Faculty of Sciences, Catholic University of the North, 0610-Antofagasta, Chile; E-Mail: luisbustosg@gmail.com; 4 Department of Parasitology, Faculty of Pharmacy, Complutense University of Madrid (CEI Campus Moncloa, UCM-UPM & CSIC), 28040-Madrid, Spain; E-Mails: crfonseca@pdi.ucm.es (C.F.-B.); agbarrio@ucm.es (A.G.-B.); 5 Research Center on Health for the Tropics, (CIST), Carretera Troncal del Caribe, Sector Mamatoco, Santa Marta, Magdalena, Colombia; E-Mail: juandib@hotmail.com

**Keywords:** aminoalcohols, diamines, *Trypanosoma cruzi*, epimastigotes, amastigotes, cytotoxicity

## Abstract

Thirteen aminoalcohols and eight diamines were obtained and tested against *Trypanosoma cruzi* epimastigotes strains MG, JEM and CL-B5 clone. Some of them were equal or more potent (1.0–6.6 times) than the reference compound nifurtimox. From them, three aminoalcohols and two diamines were selected for amastigotes assays. Compound **5** was as potent as the reference drug nifurtimox against amastigotes of the CL-B5 strain (IC_50_ = 0.6 µM), with a selectivity index of 54.

## 1. Introduction

Chagas disease or American trypanosomiasis is a zoonotic infection caused by the hemoflagellate protozoan parasite *Trypanosoma cruzi.* Infection is transmitted to humans by bites and concomitant defecation of different triatomine species, which carry the parasite in their contaminated faeces. In endemic areas, the main mode of infection is vectorial, by domestic, peridomestic, or sylvatic triatomines. However, the existence of non-vectorial mechanisms of transmission (*i.e.*, blood transfusion, organ transplant or congenital infection) has contributed in the spread of the disease to non-endemic areas, such as United States, Canada, Japan, Australia or European countries [1,2]. The disease affects millions of people generating health, economic and social problems in the countries affected [3]. It is widespread in Central and South America, affecting 21 countries in these regions. It has been estimated that the disease affects between 9.8 and 11 million people, while 60 million are at risk. This is due to residence in endemic areas and population mobility between Latin-American countries and the rest of the world.

In most patients the infection is asymptomatic or shows mild symptoms. Over a period of decades, 15%–30% of them will develop a chronic stage with cardiac or gastrointestinal clinical manifestations. In addition, Chagas disease is now seen as an opportunistic infection in immunocompromised patients with reactivation syndromes as acute myocarditis or meningoencephalitis [4,5]. The only available drugs for the etiological treatment of Chagas disease, nifurtimox (NFX) and benznidazole (Bz), are very old and cause severe side effects. They produce an average cure rate of 80% in acute cases, and less than 20% in chronic cases. NFX causes gastrointestinal side effects in 30%–70% of patients, irritability, insomnia and mood changes. Bz causes up to 30% mild to moderate dermatitis in adults. Also, digestive, hepatic, peripheral nerve and bone marrow disorders and teratogenicity have been reported. Resistance and cross-resistance to both drugs have also been observed [6,7,8,9].

A great number of natural and synthetic compounds have been tested in the past few years as trypanocidal, including drugs uses in the market for the treatment of certain diseases as antiarrhythmics [10] or antifungals [11] among others. Sphingosine is a natural unsaturated C-18 linear aminodiol, which mainly integrates sphingolipids, ceramides and other lipidic metabolites. Sphingosine-1-phosphate has been recognised as a multifunctional physiologic mediator, with roles in regulating cardiogenesis, vascular system formation, oocyte survival and immune cell trafficking. It has also been proposed as an intracellular second messenger [12] and, among other functions, is able to balance and control the levels of sphingolipid metabolites and, consequently, to regulate signalling pathways, determining whether the cell survives or dies. Sphingosine, its metabolites and their derivatives have been considered as target molecules for the design of potential candidates in the development of new drugs [13]. In fact, several reports describe antimicrobial, antiparasitic and other pharmacological properties for linear or substituted aminoalcohols, aminodiols and diamines [14,15,16].

**Figure 1:**
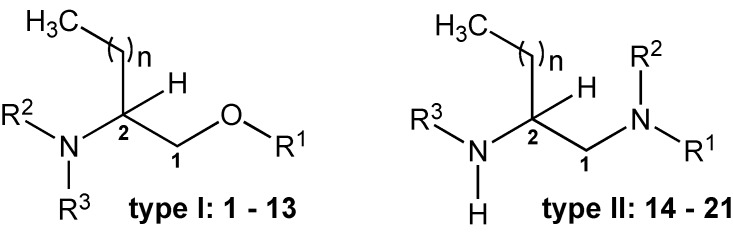
General structure of compounds type I and II.

The CIETUS-Pharmaceutical Chemistry group (PharmChem) has been working on the design, synthesis and biological evaluation of lipidic molecules related to dihydrosphingosine, particularly 2-aminoalkanols and 2-aminocycloalkanols, and has reported some results on their leishmanicidal [17,18], trypanocidal [18,19], and antimycobacterial [20] activities, among others. In the present study the CIETUS-PharmChem and Parasitology groups, in collaboration with the UCM Parasitology group have synthesised and evaluated *in vitro* the activity of some new aminoalcohol and diamine derivatives (Figure 1), with different chain-length and substituents sizes, against epimastigotes and amastigotes of *T. cruzi*. The cytotoxic effect of the compounds on J774.2 mouse macrophages has also been assessed.

## 2. Results and Dicussion

### 2.1. Chemistry

**Scheme 1:**
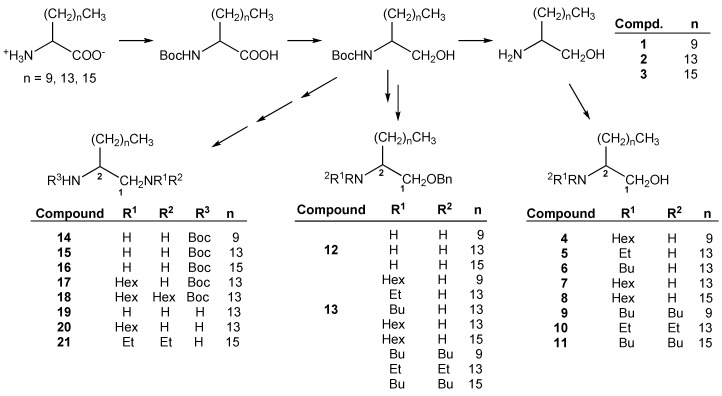
Summary of the synthetic procedure for obtaining type I compounds **1**–**13** and type II ones **14**–**21**.

The general procedures for the synthetic preparation of 2-aminoalkan-1-ol (type I compounds) and 1,2-diamine (type II compounds) derivatives were previously reported by the CIETUS-PharmChem group [17,19,20]. All the compounds were obtained and assayed as racemic mixtures. Briefly (Scheme 1), the chemical procedure started with the preparation of three long-chain α-amino acids with three common but different sizes (2-aminolauric, 2-aminopalmitic and 2-aminostearic acids). The amino group was protected as a *tert*-butyl carbamate (Boc) and the acid group reduced to the corresponding alcohol, through their mixed anhydrides [18].

On the one hand, the Boc protecting group was removed by treatment with trifluoroacetic acid to give the free amine, compounds **1**, **2** and **3**. Then, the 2-aminoalkan-1-ols 1, 2 and 3 were alkylated either with ethyl, *n*-butyl or *n*-hexyl bromide in the presence of patassium carbonate to provide the secondary amines, compounds **4**–**8**, or the tertiary amines, compounds **9**–**11**. On the other hand, the primary alcohol of the intermediate Boc-aminoalkanols was converted into a benzyl ether, then the Boc protecting group removed to give the free amine, which was further alkylated (ethyl, *n*-butyl, *n*-hexyl). Only two benzyl ether compounds (**12** and **13**, Scheme 1) were included in the antiparasitic assays to compare the activity with respect to the free alcohols. 1,2-Diamine derivatives were obtained from the Boc-amino alkanols, by successive conversion of the alcohol group into a mesylate and an azide, whose reduction generated the corresponding *N*^2^-Boc-diamines. Then, the free primary amine at position C-1 was conveniently alkylated (ethyl, *n*-hexyl) to give compounds **14** to **18**. Finally, the removal of the protecting carbamate group by hydrolysis in acidic medium generated the diamines **19** to **21**. All the compounds obtained were conveniently characterized through their IR, MS, ^1^H-NMR and ^13^C-NMR spectral data.

### 2.2. Biological Activity

The antitrypanocidal capacity of the thirteen β-aminoalcohol and eight ethylenediamine derivatives with different chain lengths (lauryl, palmityl and stearyl, *n* = 9, 13 and 15, respectively), was measured with two techniques, the Trypan-Blue and 2,3-bis-(2-methoxy-4-nitro-5-sulfophenyl)-5-[(phenyl-amino)carbonyl]-2*H*-tetrazolium hydroxide (XTT) assay. Concentrations ranging 0.1–70.0 µM were tested as described in experimental section. The activity results expressed as IC_50_ (Mean ± SEM) against *T. cruzi* epimastigote strains MG and JEM, are presented in Tables 1 (type I: aminoalcohol derivatives **1**–**13**) and Table 2 (type II: diamine derivatives **14**–**21**). Nifutimox Index (NI) and Selectivity Index (SI) were used to compare with nifutimox activity and macrophage toxicity. The general structures of the compounds are indicated in Figure 1.

Comparing the aminoalcohols and aminobenzyl ethers (type I) included in Table 1, it can be observed that against the MG strain, compounds **6**, **7** and **1** resulted more potent than NFX and compounds **4**, **5** and **2** appeared as potent as the reference drug. Among them, the least toxic were compounds **6** (SI = 20) and **2** (SI = 3.3). From the comparison of compounds **1**, **2** and **3**, with unsusbstituted primary amine and alcohol groups, the *n*-decyl chain (*n* = 9) was deduced as the best for the activity, compound **1** being the most potent (1.5 times NFX) though compound **2** the less toxic (SI = 3.3). Similar comparison and deductions can be made from compounds **4**, **7** and **8** (*n*-hexylated secondary amine and free alcohol group), in this case better activity was found for compound **7**, with an *n*-tetradecyl chain (n = 13), unluckily, compound **7** was found to be the most toxic of the three. Interestingly, compound **7** displayed 11 times greater activity on the MG strain (IC_50_ = 6 µM) than the JEM strain (IC_50_ >70 µM), while conversely the MG strain resulted >40% less susceptible than to NFX the JEM strain (Table 1). The influence of akylation of the amino group on the trypanocidal activity can be assessed through comparison of the results for the secondary amines of the palmityl series **2**, **5**, **6** and **7** without substitution and with ethyl, *n-*butyl and *n-*hexyl groups, respectively. As it is observed, within the limited number of substituents checked, the *N*-alkylation increases the trypanocidal potency with the highest influence for the *n*-butyl group, compound **6** is 6.6 times more potent and 1.3 times less toxic than NFX. Among the tertiary amines, compounds **9**, **10** and **11**, only compound **9** (*N*,*N*-dibutylamine, and *n* = 9) was active, being 0.5 times less potent but at the same time 1.3 times less toxic than NFX. Benzylation of the alcohol function (compounds **12** and **13**) gave less active and more toxic compounds, as can be deduced by comparing compounds **2** (free alcohol) and **12** (benzyl ether).

**Table 1:** *In vitro* activity of aminoalcohol derivatives against epimastigotes of two strains of *Trypanosoma cruzi*. 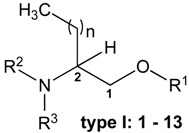

Compound	R^1^	R^2^	R^3^	*n*	MG Strain			JEM Strain			Macrophages
					IC_50_ (µM)	P_NFX_	SI	IC_50_ (µM)	P_NFX_	SI	LC_50_ (µM)
**1**	H	H	H	9	**14.5 ± 3.15**	**1.5**	1.0	33.0 ± 1.70	0.4	0.4	14.0 ± 0.90
**2**	H	H	H	13	23.7 ± 3.15	0.9	**3.3**	3.0 ± 1.50 *	**4.9**	**26**	**77.8 ± 0.35**
**3**	H	H	H	15	46.2 ± 2.50	0.5	0.1	9.9 ± 1.65 *	**1.5**	0.3	3.0 ± 0.24
**4**	H	H	Hex	9	**21.1 ± 1.25**	**1.0**	1.7	37.2 ± 1.25	0.4	1.0	36.1 ± 2.30
**5**	H	H	Et	13	**21.6 ± 2.95**	**1.0**	2.0	19.3 ± 1.35	0.8	2.3	43.4 ± 0.50
**6**	H	H	Bu	13	3.2 ± 0.20 *	**6.6**	**20**	2.8 ± 0.75 *	**5.3**	**23**	**63.9 ± 3.50**
**7**	H	H	Hex	13	6.1 ± 0.15 *	**3.5**	1.3	*na*	-	-	8.2 ± 1.55
**8**	H	H	Hex	15	*na*	*-*	*-*	*na*	-	-	26.0 ± 0.20
**9**	H	Bu	Bu	9	39.2 ± 0.20	0.5	1.6	50.5 ± 0.70	0.3	1.3	**63.7 ± 0.73**
**10**	H	Et	Et	13	*na*	*-*	*-*	*na*	-	-	5.6 ± 0.27
**11**	H	Bu	Bu	13	*na*	-	-	50.3 ± 0.10	0.3	-	*nt*
**12**	Bn	H	H	13	34.4 ± 1.80	0.6	0.8	28.8 ± 1.20	0.5	1.0	27.7 ± 0.10
**13**	Bn	H	Bu	13	*na*	*-*	*-*	*na*	*-*	*-*	*nt*
**NFX**					21.2 ± 1.16	1.0	2.3	14.8 ± 1.24	1.0	3.3	49.1 ± 1.15

P_NFX_ (potency relative to NFX) = IC_50_-NFX/IC_50_-compound. SI (Selectivity Index): LC_50_-macrophages/IC_50_-epimastigotes. *na*: >70 µM. Bn: benzyl; Bu: *n*-butyl; Hex: *n*-hexyl; Et: ethyl; * statistical significance (*p* < 0.05). Results better than those found for NFX are **bold-faced** for easier comparison.

A similar structure activity relationship (SAR) analysis can be performed with the results found against the more sensitive JEM strain for these β-aminoalcohol derivatives. The most remarkable differences lie on the increased potency and selectivity for compound **2** (2-aminohexadecanol, IC_50_ = 3.0 µM, NI = 4.9, SI = 26), that approaches the activity of this compound to that of compound **6** (2-butylaminohexadecanol), the most potent compound against both strains. The last showed low-µM IC_50_ values of statistical significance (*p* = 0.004), with NI values of 6.6 and 5.3 times more potent than NFX against the MG and JEM strain, respectively, in a dose-dependent manner, and with selectivity indexes SI of 20 or higher, that are substantially better than those found for NFX. In order to complete the SAR analysis for this group of compounds it should be noted that dialkylation of the amino group (tertiary amines **9**, **10** and **11**) or benzylation of the alcohol function (compounds **12** and **13**) resulted in less potent or even inactive (IC_50_ > 70 µM) compounds.

The most interesting compounds **2** and **6** are structurally very close, and also recognized as β-aminopalmitol (the reduction product of α-aminopalmitic acid) and β-(butylamino)palmitol, respectively. Their trypanocidal activity and their lesser toxicity values for macrophages, with SI values substantially better than those found for NFX (2.3 and 3.3 in those experiments performed in this research, Table 1) led to their selection for further research. 

The eight diamine derivatives **14** to **21** were also tested *in vitro* against epimastigotes of both MG and JEM strains of *T. cruzi*, and the results are shown in Table 2. Globally, they resulted less potent and selective than the aminoalcohol derivatives discussed above, though several of them attained and surpassed those levels of activity (up to the double for the MG strain by **14**, and to 2.2 times for the JEM strain by **16**) and selectivity (SI = 4.4 on MG for **14** and 3.6 on JEM for **16**) found for NFX. The most potent compounds for both strains were the Boc-monoprotected diamines **14**, **15** and **16**. Related to the influence of the chain size of diamines on the activity, results in Table 2 indicate the same, though softer, tendency as the aminoalcohols against the MG strain. A decrease of trypanocidal potency with the increasing of chain size (NI: 1.5 for the C_18_-stearyl derivative **16**, 1.8 for the C_16_-palmityl derivative **15**, and 2.0 for the C_12_-lauryl compound **14**). The softer influence of the chain size on the activity, in comparison with the case of aminoalcohols, could be associated to the presence of the Boc-protector, which through its electron-attracting carbamate function would modulate, disturb or even prevent the interaction of the amino group at C-2 with the target molecule in the parasite. On the other hand, it must be noted that this not certain in the case of the JEM strain, where the best trypanocidal response is observed for the C_18_-stearyl derivative 16 (2.2 times more active than NFX**)**. Respecting the influence of the Boc-carbamate group attached to the *N*^2^ atom, the comparison of results found for the Boc-compounds **15** and **17** (NI: 1.8 and 0.8 for MG; 0.7 and 0.5 for JEM strains, respectively) with those for the unprotected compounds **19** and **20** (NI: 1.1 and 0.6 for MG; 0.6 and 0.3 for JEM strains, respectively), all of them with the same palmityl chain size, allows to recommend the presence of the Boc-carbamate or a similar group to reinforce the activity of diamines. Finally, related to the effect of amine alkylation, and due to the predominant presence of the Boc-protecting group at *N*^2^, only mono and dialkylation on *N*^1^ is being considered. Dialkylation was clearly negative, as can be observed for the tertiary dihexylamine **18** (IC_50_ > 70 µM against both strains) and for the diethylamino analogue **20** (IC_50_ = 42.2 against MG and >70 µM against JEM). Similarly, the effect of monoalkylation can be deduced as negative, from comparison of the compounds pairs 15 *vs.* 17 and **19** *vs.* **20** with the same palmityl chain. As a consequence, along with the presence of the Boc group at *N*^2^, the nature of primary amine at C-1, was recommended for a higher trypanocidal activity in this type of compounds. Accordingly, those Boc-diamines **14** and **15**, displaying the highest P_NFX_ values against the less sensitive MG strain, and the aminoalcohol derivatives **2**, **5** and **6** were selected for further evaluation.

The five compounds were then evaluated against epimastigotes and amastigotes of *T. cruzi* (CL Brener strain clone B5, CL-B5) and the trypanocidal activity results based in β-galactosidase activity are shown in Table 3*.* P_NFX_ also used to compare compound potency with nifurtimox. Cytotoxicity assays on NCTC-929 fibroblasts cells were also performed.

**Table 2:** *In vitro* activity of diamine derivatives against epimastigotes of two strains of *Trypanosoma cruzi*. 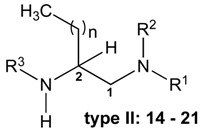

Compound	R^1^	R^2^	R^3^	*n*	MG Strain			JEM Strain			Macrophages
					IC_50_ (µM)	P_NFX_	SI	IC_50_ (µM)	P_NFX_	SI	LC_50_ (µM)
**14**	H	H	Boc	9	10.8 ± 0.0 *	**2.0**	**4.4**	**14.5 ± 0.05**	**1.0**	**3.3**	**47.1 ± 0.35**
**15**	H	H	Boc	13	**12.0 ± 2.15**	**1.8**	**4.0**	20.4 ± 0.30	0.7	2.4	**48.1 ± 0.15**
**16**	H	H	Boc	15	**13.9 ± 1.00**	**1.5**	1.7	**6.7 ± 2.10**	**2.2**	**3.6**	24.1 ± 0.20
**17**	Hex	H	Boc	13	26.7 ± 0.25	0.8	0.2	30.6 ± 0.65	0.5	0.1	4.2 ± 0.05
**18**	Hex	Hex	Boc	13	*na*	*-*		*na*	*-*	*-*	*nt*
**19**	H	H	H	13	**19.2 ± 2.90**	**1.1**	**4.1**	26.6 ± 0.70	0.6	2.9	**78.1 ± 0.85**
**20**	Hex	H	H	13	32.9 ± 1.25	0.6	0.2	48.8 ± 1.05	0.3	0.1	5.0 ± 0.10
**21**	Et	Et	H	13	42.2 ± 1.4	0.5	0.2	*na*	-	-	9.3 ± 0.50
**NFX**					21.2 ± 1.16	1.0	2.3	14.8 ± 1.24	1.0	3.3	49.1 ± 1.15

P_NFX_ (potency relative to NFX) = IC_50_-NFX/IC_50_-compound. SI (Selectivity Index): LC_50_-macrophages/IC_50_-epimastigotes. *na*: >70 µM. *nt*: Non tested. Boc: *t-*butoxycarbonyl; Hex: *n*-hexyl; Et: ethyl; * statistical significance (*p* < 0.05). Results better than those found for NFX are **bold-faced** for easier comparisons.

All the compounds were more potent than NFX against the epimastigote form with P_NFX_ values ranging from 2.5 to 4.3, whereas only the aminoalcohol **5** attained the potency of NFX against amastigotes. 

**Table 3:** *In vitro* activity against epimastigotes and amastigotes of *Trypanosoma cruzi* CL-B5 clone and NCTC-929 cytotoxicity values of selected aminoalcohol and diamine derivatives. 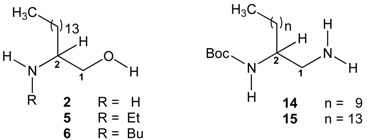

Compound	Epimastigotes			Amastigotes			Cytotoxicity
	CL-B5 Clone			CL-B5 Clone			NCTC-929
	IC_50_ (µM)	P_NFX_	SI	IC_50_ (µM)	P_NFX_	SI	LC_50_ (µM)
**2**	3.5 ± 0.01 *	**3.0**	7.2	1.8 ± 0.12	0.3	14.0	25.2 ± 0.12
**5**	3.3 ± 0.10 *	**3.1**	9.8	**0.6 ± 0.25**	**1.0**	**54.0**	32.4 ± 0.02
**6**	4.2 ± 0.52 *	**2.5**	**22.7**	2.0 ± 0.27	0.3	**47.8**	95.6 ± 1.19
**14**	2.5 ± 0.03 *	**4.2**	11.8	3.0 ± 0.21	0.2	9.9	29.6 ± 0.01
**15**	2.4 ± 0.01 *	**4.3**	10.6	2.6 ± 0.23	0.2	9.8	25.6 ± 0.06
**NFX**	10.4 ± 1.3	1.0	6.7	0.6 ± 0.01	1.00	116.0	69.6 ± 0.17

Nifurtimox Index (NI): nifurtimox IC_50_/compound IC_50_. Selectivity Index (SI): NCTC-29 IC_50_/epimastigote or amastigotes IC_50_. * statistical significance (*p* < 0.05). Most significant values of potency and/or selectivity are bold faced.

Moreover, the aminoalcohols **2**, **5** and **6** resulted more active on the intracellular parasite than over the extracellular one, especially compound **5** (IC_50_ = 3.3 µM in epimastigotes *vs.* IC_50_ = 0.6 µM in amastigotes), as potent as the reference drug NFX. As for cytotoxicity, compound **6** was less toxic than NFX, while compounds **2**, **5**, **14** and **15** were slightly more toxic (2.1–2.7) than NFX on NCTC-929 fibroblasts cells (Table 3). Compound **5** was as potent as NFX on amastigotes but displayed twice the toxicity on NCTC-929 fibroblasts. The structure of compound **5**, in comparison with compound **6**, the most potent aminoalcohol in Table 1, indicates the convenience of the smaller ethyl group, rather than the butyl, attached to the amino group at C-2. The diamine derivatives, compounds **14** and **15**, against amastigotes were less potent than its aminoalcohol analogues.

## 3. Experimental Section

### 3.1. General Information

All commercial chemicals, reagents and solvents used were reagent grade. Flash column chromatography was done using Merck Silica Gel 60 (0.04–0.063 mm). Reactions were monitored by TLC using Merck 60F_254_ silica gel plates. Compounds were detected visually under UV irradiation (254 nm) and by spraying with sulfuric acid and phosphomolybdic acid reagents followed by heating at 100 °C. ^1^H-NMR and ^13^C-NMR spectra were obtained with a Bruker AC 200 spectrometer (200 and 50.3 MHz, respectively). Chemical shifts were recorded in parts per million (ppm, δ) and were re-ported relative to the solvent peak or TMS. High resolution mass spectra (HRMS) were measured with a QSTAR XL quadrupole time-of-flight mass spectrometer, by direct injection on the sample dissolved in MeOH and Ionization voltage of 5500 V. Infrared (IR) spectra were measured on a Nicolet Impact 410 spectrophotometer. The biological material was maintained in culture medium until use. Fetal calf serum (FCS), Basal Medium Eagle (BME) without phenol-red, Dulbecco’s modified essential medium (DMEM), hemin, gentamycin, EDTA-trypsin, dimethyl sulfoxide (DMSO), XTT and trypan blue 0.4% were purchased from Sigma (St. Louis, MO, USA). Glutamine, penicillin 100 U/mL, streptomycin 100 µg/mL and phosphate-buffered saline solution (PBS) were purchased from Gibco (Grand Island, NY, USA). Forty-eight-well plates and 96-well flat bottoned plates were purchased from Costar (Corning Corp, Cambridge, UK). β-Galactosidase substrate chlorophenol β-d-galactopyranoside (CPRG) were purchased from Roche Life Sciences (Indianapolis, IN, USA). Triton X-100 was purchased from Panreac (Barcelona, Spain). Liver-infusion broth and tryptose broth (LIT) growth medium were purchased from Difco (Becton Dickinson, Le Pont-de-Claix, France). Nifutimox (Lampit) was provided by Bayer (Leverkusen, Germany). The results were acquired from each test well using ELISA reader Ear400FT spectrophotometer (STL Lab Instruments, Groding, Austria).

### 3.2. Chemistry

The general method for the preparation of 2-aminoalkan-1-ol (type I compound) and 1,2-diamine (type II compound) derivatives was previously reported by the CIETUS-PharmChem group [11,19,20] and is indicated in Scheme 1. All the compounds were obtained and assayed as racemic mixtures. Here will describe the last step for obtaining compounds **2**, **5**, **6**, **14** and **15**.

#### 3.2.1. 2-Aminohexadecanol (**2**) 

Compound **2** was obtained from 2-[(*tert*-butoxycarbonyl)amino]hexadecanol by removal of the *tert*-butoxycarbonyl protecting group by treatment with trifluoroacetic acid. 2-[(*tert*-Butoxycarbonyl)amino]hexadecanol (300 mg, 0.84 mmol) was dissolved in a solution of 50% trifluoroacetic acid in dichloromethane (10 mL) and the mixture then maintained 4 h at 0 °C under stirring. Reaction progress was controlled by TLC. Then, 6 M NaOH was added to neutral pH and the mixture was extracted with dichloromethane (3 × 40 mL). The organic layer was washed with water and dried over Na_2_SO_4_. The solvent was removed under vacuum to give 186 mg (93%) of **2** as a white solid, m.p. = 66–67 °C; IR: 3420, 3368, 2922, 2852, 1568, 1477, 1388, 1079 cm^−1^; ^1^H-NMR (CDCl_3_): δ 0.87 (3H, t, *J* = 6.5 Hz, H-16), 1.25 (26H, br s)*,* 2.78 (1H, m, H-2), 3.24 (1H, dd, *J* = 10.4; 7.5 Hz, H-1a), 3.54 (1H, dd, *J* = 10.4; 3.6 Hz, H-1b); ^13^C-NMR (CDCl_3_): δ 14.1, 22.7, 26.2, 29.4, 29.6 (7C), 32.0, 34.1, 52.8, 65.7; HRMS (ESI^+^) for C_16_H_36_NO [M + H]^+^: calcd: 258.2797; found: 258.2801.

#### 3.2.2. 2-(Ethylamino)hexadecanol (**5**)

2-Aminohexadecan-1-ol (50 mg, 0.19 mmol) was added to a suspensión of K_2_CO_3_ (52 mg, 0.19 mmol) in DMF (2 mL) at room temperature under stirring, and the mixture was stirred for another 15 min. Then, ethyl bromide (28 μL; 0.38 mmol) was added and the mixture maintained for 15 h at room temperature. Reaction progress was controlled by TLC. Ethyl acetate (40 mL) was added, and the organic layer washed with water to neutral pH. The organic layer was dried over Na_2_SO_4_, and the solvent removed under vacuum to give a crude that was purified by column chromatography with CH_2_Cl_2_/CH_3_OH (9:1), to give 34 mg (58%) of the corresponding tertiary amine and 23 mg (40%) of **5** as a white solid, m.p. = 66–67 °C; IR: 3322, 2922, 2847, 1460, 1379, 1128, 1078 cm^−1^; ^1^H-NMR (CDCl_3_): δ 0.85 (3H, t, *J* = 6.5 Hz, H-16), 1.10 (3H, t, *J* = 7.0 Hz, H-2ʹ), 1.22 (26H, br s), 2.63 (2H, m, H-1ʹ), 3.11 (1H, m, H-2), 3.29 (1H, dd, *J* = 11.0; 6.5 Hz, H-1a); 3.61 (1H, dd, *J* = 11.0; 4.0 Hz, H-1b); ^13^C-NMR (CDCl_3_): δ 14.1, 15.2, 22.7, 26.2, 29.7 (9C), 31.3, 32.0, 41.2, 59.0, 62.7; HRMS (ESI^+^) for C_18_H_40_NO [M + H]^+^: calcd: 286.5163; found: 286.5148.

#### 3.2.3. 2-(Butylamino)hexadecanol (**6**)

2-Aminohexadecan-1-ol (200 mg, 0.99 mmol) was added to a suspensión of K_2_CO_3_ (273 mg, 1.98 mmol) in DMF (3 mL), and the mixture stirred for 15 min. Then, butyl bromide (217 μL, 1.98 mmol) was added and the mixture maintained 15 h at room temperature. Reaction progress was monitored by TLC. The reaction was worked up as mentioned above for compound **5**, to give 140 mg (54%) of the corresponding tertiary amine and 23 mg (39%) of **6** as a white solid, m.p. = 54–55 °C; IR: 3431, 3292, 2922, 2848, 1465, 1374, 1063 cm^−1^; ^1^H-NMR (CDCl_3_): δ 0.87 (3H, t, *J* = 6.8 Hz, H-16), 0.91 (3H, t, *J* = 6.8 Hz, H-4ʹ), 1.25 (26H, br s), 2.54 (2H, m, H-1ʹ), 2.68 (1H, m, H-2), 3.27 (1H, dd, *J* = 10.4; 6.4 Hz, H-1a); 3.62 (1H, dd, *J* = 10.4; 3.9 Hz, H-1b); ^13^C-NMR (CDCl_3_): δ 14.0, 14.2, 20.5, 22.7, 26.3, 29.3, 29.7 (8C), 31.5, 32.0, 32.4, 46.5, 59.1, 62.8; HRMS (ESI^+^) for C_20_H_44_NO [M + H]^+^: calcd: 314.3423; found: 314. 3417.

#### 3.2.4. 2-(*tert-*Butoxycarbonyl)-dodecan-1,2-diamine (**14**) 

2-[(*tert*-Butoxycarbonyl)amino]dodecanol (500 mg, 1.66 mmol) was dissolved in dichloromethane (3 mL) and the solution maintained at 0 °C under stirring. Then, triethylamine (448 μL, 1.66 mmol) and mesyl chloride (448 μL, 1.66 mmol) were added and the mixture kept at 0 °C for 30 min, followed by 30 min a room temperature. Reaction progress was followed by TLC. After that, dichloromethane (80 mL) was added and the organic layer washed with saturated sodium chloride, 2 N HCl and water solutions to neutral pH. The organic layer dried over Na_2_SO_4_, filtered and concentrated under vacuum to provide 673 mg (99%) of the mesylate derivative. The obtained mesylate derivative was dissolved in dimethyl formamide (DMF, 5 mL) and sodium azide (335 mg, 5.30 mmol) was added under stirring. The mixture was heated at 50–60 °C for 6 h. DMF was removed under high pressure and the resulting residue redissolved in ethyl acetate (100 mL) and washed with water. The organic layer was dried over Na_2_SO_4_, filtered and concentrated under vacuum to provide 900 mg of a crude product that was purified by column chromatography to provide 710 mg (65%) of the azido derivative. The obtained azido derivative was dissolved in dry tetrahydrofuran (THF, 4 mL) and NaBH_4_ (125 mg, 1.00 mmol) was added, followed by the addition of methanol (30 mL, dropwise from an addition funnel) for 30 min, under stirring. Then, ethyl acetate (100 mL) was added and the organic layer washed with water, dried over Na_2_SO_4_, filtered and concentrated under vacuum to provide 660 mg of a crude that was purified by column chromatography to provide 236 mg (76%) of **14** as an oil with the following properties: IR: 3362, 2925, 2855, 1689, 1524, 1388, 1367, 1250, 1173, 1054, 894 cm^−1^; ^1^H-NMR (CDCl_3_): δ 0.87 (3H, t, *J* = 6.8 Hz, H-12), 1.25 (18H, br s)*,* 1.44 (9H, s, (CH_3_)_3_), 2.61 (1H, dd, *J* = 12.9; 6.8 Hz, H-1a), 2.78 (1H, dd, *J* = 12.9; 4.3 Hz, H-1a), 3.52 (1H, m, H-2), 4.53 (1H, d, *J* = 9.3 Hz, NH); ^13^C-NMR (CDCl_3_): 14.1, 22.7, 26.0, 28.4, 29.4, 29.6, 30.5, 31.9, 32.9, 46.2, 53.2, 79.1, 156.1; HRMS (ESI^+^) for C_17_H_36_N_2_O_2_Na [M+Na]^+^: calcd. 323.2674; found. 323.2651.

#### 3.2.5. 2-*tert*-Butoxycarbonyl-hexadecan-1,2-diamine (**15**)

Compound **15** was obtained by applying the same procedure. Starting from 2-[(*tert*-butoxy- carbonyl)amino-hexadecanol (1.00 g) 578 mg (56% global yield) of **15** was obtained as a white solid with m.p. = 45–46 °C; IR: 3375; 2922; 2852; 1709; 1547; 1386; 1367; 1176; 1052; 893 cm^−1^; ^1^H-NMR (CDCl_3_): δ 0.87 (3H, t, *J* = 6.8 Hz, H-12), 1.24 (26H, br s)*,* 1.44 (9H, s, (CH_3_)_3_), 2.60 (1H, dd, *J* = 12.8; 6.8 Hz, H-1a), 2.76 (1H, dd, *J* = 12.8; 4.6 Hz, H-1a), 3.49 (1H, m, H-2), 4.52 (1H, d, *J* = 8.6 Hz, NH); ^13^C-NMR (CDCl_3_): δ 14.2, 22.7, 26.0, 28.4, 29.4, 29.7, 31.7, 33.0, 46.2, 53.2, 79.5, 156.1; HRMS (ESI^+^) for C_21_H_44_N_2_O_2_Na [M+Na]^+^: calcd. 379.3300; found. 379.3295.

### 3.3. In Vitro Assays

#### 3.3.1. Parasite Strains, Culture and Assays Procedures

For *in vitro* studies, the strains MHOM/CO/04/MG (MG), MHOM/CO/05/JEM (JEM) and CL Brener strain clone B5 (CL-B5) were used. The MG and JEM strains correspond to Group I and Haplotype Ia isolates from two Colombian patients in the acute phase [21], and CL-B5 is a strain stably transfected with *Escherichia coli* β-galactosidase gene (lacZ) [22]. Epimastigotes were grown in plastic culture flasks at 28 °C, in axenic liver-infusion tryptose (LIT) growth medium, supplemented with 10% heat inactivated (30 min at 56 °C) foetal calf serum (FCS; Sigma), 25 mg/mL hemin (Sigma), and 10 µg/mL gentamycin (Sigma) and harvested in the exponential phase for conducting the experiments. Cultures were continuously maintained in logarithmic growth by weekly passages.

For the anti-amastigote assays, axenic cultures of epimastigotes reaching their stationary phase of growth (14 day-old cultures) were used to infect non-confluent monolayers of NCTC-929 fibroblasts to obtain tissue culture derived trypomastigotes (TCT). After 24 h at 33 °C in a humidified 5% CO_2_ atmosphere, the infected cultures were washed with phosphate buffer saline (PBS) to removed non-penetrated epimastigotes. TCT were harvested from the supernatant of the cultures after one week of incubation in these conditions of temperature and humidity. 

#### 3.3.2. Macrophage and Fibroblast Cultures

The mouse cell lines J774.2 macrophages and NCTC clone 929 fibroblasts were used and cultured in plastic culture flasks. J774.2 and NCTC-929 cells were grown using DMEM and BME without phenol-red, respectively. Both media were supplemented with 10% heat inactivated fetal calf serum, 2 mM glutamine, 100 U/mL penicillin and 100 μg/mL streptomycin. Cell cultures were maintained at 37 °C in a humidified atmosphere containing 5% CO_2_ and subpassaged once a week. Cells in the pre-confluence phase were harvested with an EDTA-trypsin solution for the experiments. 

#### 3.3.3. Epimastigote Susceptibility Assay

Three different strains of *T. cruzi* were used for the primary screening on epimastigote forms. For strains MG and JEM, the screening assay was performed with approximately 1 × 10^5^ epimastigotes in logaritmic growth phase in 200 μL of supplemented LIT medium per well in 96-well flat bottoned plates (Costar, Corning, Cornig, NY, USA). Stock solutions of the compounds were prepared in dimethyl sulfoxide (DMSO) (Panreac) to obtain final concentrations of 0.1, 0.5, 3.3, 15.0, 35.0 and 70.0 μM and the concentration of DMSO in cultures was always lower than 0.2% (*v*/*v*). Mortality control with the reference drug nifurtimox (Bayer) was included in the assay. The plates were incubated for 72 h at 28 °C and all experimental conditions were evaluated in triplicate in three independent experiments. The epimastigote growth inhibition was microscopically determined by the Trypan Blue dye exclusion assay and XTT (2,3-bis-(2-methoxy-4-nitro-5-sulfophenyl)-5-[(phenylamino)carbonyl]-2*H*-tetrazolium hydroxide). Regarding the first method, an aliquot from every culture was suspended in 0.4% trypan blue solution for 5 min and cell viability was determined under microscope by counting live parasites in each condition in a Neubauer chamber [23]. Anti-epimastigote activity percentage was calculated as (Parasite counts in control wells-Parasite counts in experimental wells) × 100/Parasite counts in control wells. Trypanocidal activity over both strains was also measured adding 50 μL of XTT solution per well to obtain a final concentration of 0.3 mg/mL. The plate was re-incubated 24 h in the dark at 37 °C and orange formazan solution formed, was spectrophotometrically quantified at 450 nm with a 630 nm reference filter, using an Ear400FT ELISA reader.

Trypanocidal activity against CL-B5 strain was also assessed by measuring β-galactosidase activity. Briefly, log-phase epimastigotes were seeded in 96-well microplates at a density of 2.5 × 10^5^ parasites/mL in a final volume of 200 μL/well and incubated within the compounds for 72 h at 28 °C. Afterwards, 50 μL of the β-galactosidase substrate chlorophenol red β-d-galactopyranoside (CPRG, Roche) in 0.9% Triton X-100 (Panreac) solution were added to each well (200 μM, pH 7.4) and the plates incubated at 37 °C for 3 h. Anti-epimastigote activity was estimated by reading absorbance at 595 nm. Each concentration was assayed by triplicate, in three independent experiments. The reference drug nifurtimox (Lampit) was also tested. In all spectrophotometric assays, anti-epimastigote activity percentages were calculated as (OD control wells − OD experimental wells) ×100/OD control wells.

The antiparasitic activity of compounds **1**–**21** was expressed as the concentration that inhibited 50% of parasites (IC_50_) and it was calculated by sigmoidal regression analysis [24]. The trypanocidal activity of those assayed compounds was compared to that of NFX using the Nifurtimox Index (NI = nifurtimox IC_50_/compound IC_50_). Furthermore the Selectivity Index (SI = mammalian cell LC_50_/epimastigote IC_50_) for each compound was calculated to compare the antiprotozoal activity with its respective mammalian cell cytotoxicity. Compounds with trypanocidal activity comparable or better than nifurtimox against MG or JEM strains were confirmed in CL-B5 epimastigote and amastigote susceptibility assays. Compounds with IC_50_ > 70 μM were considered not active (na).

#### 3.3.4. Amastigote Susceptibility Assay

For this assay, 120 μL of BME containing 10,000 NCTC-929 fibroblasts/well were seeded in 48-well plates (Costar) and after their attachment, infected with TCT obtained as above in a 1:6 cell-parasite ratio. The plates were incubated at 33 °C and 5% CO_2_ overnight and after the infection, experimental wells were washed with PBS to eliminate non-penetrated TCT. Solutions of each compound and nifurtimox were added in fresh medium (final concentrations of 0.1–70.0 μM) in a final volume of 450 μL/well and incubated for 7 days in similar conditions of temperature and humidity. All experimental conditions were evaluated by triplicate in three independent experiments and nifurtimox was tested in parallel. Then, 50 μL of a CPRG solution in 3% Triton X-100 (pH 7.4) were added to each well in a final concentration of 400 μM and the plates incubated 3 h at 37 °C. Finally, anti-amastigote activity was estimated by reading absorbance at 595 nm in a plate reader (Infinite 200, Tecan) and calculated as in Fonseca-Berzal *et al.* [25]. IC_50_ and NI indexes were calculated as above.

#### 3.3.5. Cytotoxicity Assays

Unspecific cytotoxicity was evaluated over phagocytic macrophages J774.2 cell line. To study cytotoxicity on J774.2 cells, 200 μL of a suspension of 2 × 10^5^ macrophages/mL were added to 96 well flat-bottom microplates (Sigma). Cells were allowed to attach for 24 h at 37 °C and then exposed to the compounds for 72 h at the same concentrations as used for drug screening. A solution of 0.2% DMSO and nifurtimox ranging 0.1–70 μM were used as controls [26]. Each concentration was assayed by triplicate, in independent experiments. Cytotoxicity was assessed by the Trypan Blue and XTT techniques and LC_50_ was calculated as described above.

NCTC-929 cells were placed in triplicate in 96-well microtitre plates at 15 × 10^3^ cells per well in 100 µL of MEM supplemented with FBS and antibiotics. The cells were grown overnight at 37 °C in 5% CO_2_. Thereafter, the medium was removed and the compounds were added in 200 µL of medium and plates were incubated 48 h at 37 °C in 5% CO_2_. After incubation, 20 µL of 2 mM resazurin (Sigma) solution was added to each well. The plates were incubated for 3 h and metabolic activity was measured by fluorimetric readings taken in a plate fluorometer (Infinite 200, TECAN, Mannedorf, Switzerland) at λexcitation 535 nm and λemission 590 nm, according to the Alamar blue^®^ Assay. LC_50_ was calculated for each compound and NFX.

#### 3.3.6. Statistical Analysis

The results were expressed as mean and standard error of mean (SEM). Normal distribution of data was studied by the non-parametric Kolmogorov-Smirnov test. Significant differences between groups were found using Kruskal-Wallis test or one-way ANOVA test and Tukey’s honest significance test (HSD). All statistical analyses were considered significant at the *p* < 0.05 level. IC_50_ and LC_50_ values were calculated by plotting drug concentration *vs.* antiepimastigote or antiamastigote activities.

## 4. Conclusions

In summary, the aminoalcohols **1**, **6** and **7** (IC_50_ = 14.5, 3.2 and 6.1 µM, respectively) and the diamines **14**, **15**, **16** and **19** (IC_50_ = 10.8, 12.0 and 13.9 µM and 19.2 µM, respectively) were more potent than NFX against epimastigotes of the MG strain of *T. cruzi*, while against the JEM strain the aminoalcohols **2**, **3** and **6** (IC_50_ = 3.0, 9.9 and 2.8 µM, respectively) and the diamines **14** and **16**, (IC_50_ values of 14.5 and 6.7 µM, respectively) were more effective. Our data support the difference in the susceptibility of MG, JEM strains (both Colombian strains DTU I) for compounds **2** and **6** but did not show so significant difference in susceptibility to NFX. A recent study described large variations in the response to ergosterol biosynthesis inhibitors *in vitro* assays against a panel of *T. cruzi* strains and clones belonging to different genetic lineages; in contrast, Bz and NFX did not differ significantly in their efficacy against the same panel of strains and clones [27]. Nevertheless, the available evidence supports the proposition that assessing the activity of new compounds against a representative panel of strains is essential. Therefore the aminoalcohol **6** resulted more effective and less toxic than NFX for both strains. Compounds **2**, **5**, **6**, **14** and **15** were more potent than NFX against epimastigotes of the CL-B5 strain, and compound **5** was as potent as NFX against amastigotes of the CL-B5 strain (IC_50_ = 0.6 µM), with a selectivity index of 54. This preliminary study introduce aminoalcohols and diamines as suitable templates for the design of new antichagasic prototypes. However, further *in vivo* studies on murine Chagas models need to be conducted in order to confirm the outcomes achieved *in vitro*.
